# Emergency Obstetric Hysterectomy after Conservative Management of Placenta Accreta

**DOI:** 10.1155/2023/2420333

**Published:** 2023-02-27

**Authors:** T. Loukopoulos, A. Zikopoulos, M. Plachoura, A. Galani, K. Zikopoulos, E. Kolibianakis

**Affiliations:** ^1^Department of Obstetrics and Gynecology, University Hospital of Ioannina, Ioannina, Greece; ^2^Obstetrics and Gynecology Royal Cornwall Hospital, Cornwall, UK; ^3^Aristotle University of Thessaloniki, Thessaloniki, Greece; ^4^Unit for Human Reproduction, Medical School, Aristotle University of Thessaloniki, Thessaloniki, Greece

## Abstract

**Background:**

Obstetric hemorrhage is a frequent and life-threatening complication of either vaginal or cesarean delivery. It can be due to many causes, one of which is placenta accreta, the abnormal invasion of the placenta into the myometrial wall of uterus. Ultrasonography is the first line diagnostic method that can lead to the diagnosis of placenta accreta although, the depth of penetration is estimated by magnetic resonance imaging. Placenta accreta is a life-threatening situation requiring an experienced health care team for its management. Hysterectomy is usually performed although, conservative management might be preferred in carefully selected cases. *Case Presentation.* A 32-year-old woman (G2, P0) who had an inconsistently monitored pregnancy appeared at a regional hospital with contractions at 39th week of gestation. In her first pregnancy, she was subjected to cesarean section due to delay in second stage of labor and unfortunately her child died due to sudden cardiac death. During C-section, placenta accreta was identified. Given her previous history and her desire to maintain fertility, conservative management was initially planned to preserve her uterus. However, due to persisting vaginal bleeding immediately after delivery an emergency hysterectomy was performed.

**Conclusion:**

Conservative management of placenta accreta can be considered in some special cases with the aim to spare fertility. However, if bleeding cannot be controlled during the immediate postpartum period, emergency hysterectomy is unavoidable. A specialized multidisciplinary medical team is required to optimize management.

## 1. Introduction

Postpartum hemorrhage (PPH), which is defined as the total blood loss >500 ml during vaginal delivery or less than 1000 ml during cesarean delivery accompanied by signs of hypovolemia [1] can be life-threatening. The main reasons for heavy post-partum bleeding include uterine atony, retained tissue/invasive placenta, genital tract trauma, and coagulation abnormalities [2]. Placenta accreta spectrum (PAS), also called morbidly adherent placenta—refers to a clinical condition where the placenta is not delivered spontaneously following childbirth or cannot be forcefully separated without resulting to a significant and potentially lethal bleeding [3]. Based on histological findings, it is classified into three grades: placenta accreta, where the chorionic villi are in contact with the myometrium; placenta increta, where the chorionic villi invade the myometrium; and placenta percreta, where the chorionic villi grow through the uterine serosa and may affect nearby organs [4].

There are various risk factors associated with the occurrence of PAS. A previous cesarean section is the commonest cause with the probability of PAS rising proportionally to the number of previous cesarean deliveries [5]. Women with placenta previa, especially when it overlays a uterine scar, are at an increased risk and they should be carefully assessed for the presence of PAS [6]. However, any operation that can damage the endometrium, such as myectomy, curettage, manual removal of the placenta, or uterine artery embolization [7], can potentially lead to PAS. Additional risk factors include advanced maternal age, high parity, a previous diagnosis of PAS [5], In vitro fertilization (IVF) [8], smoking [9], Asherman syndrome [10], and uterine mucosal abnormalities, such as fibroids and polyps [11].

Placenta accreta, which was once uncommon, with an estimated incidence in the 1960s of 1 in 30,000 pregnancies, is now becoming an increasingly prevalent obstetric problem. Its incidence is currently estimated at 1 in 533 pregnancies [12], which probably reflects the increase in the incidence of cesarean deliveries [13].

Antenatal detection of PAS is of great importance, since planning delivery at a tertiary hospital, well before the onset of labor or bleeding, improves outcomes for both mother and child [14]. Evidence for the presence of PAS can be recognized from the first, but it is most usually recognized in the second and third trimesters [15]. Obstetric ultrasonography is commonly used for prenatal diagnosis. The presence of placenta previa, raises the suspicion for PAS, since it is present in more than 80% of cases [14]. Multiple vascular lacunae within the placenta, loss of the normal hypoechoic zone between the placenta and myometrium, decreased retroplacental myometrial thickness and extension of the placenta into myometrium serosa, or bladder are all gray-scale abnormalities related with PAS [16]. Magnetic resonance imaging (MRI) is a second-line diagnostic method for PAS, since it is not employed as a screening method, but it can be useful to women at high risk for placenta accreta, based on ultrasonographic findings. In contrast to ultrasound, MRI can access the depth of invasion and its accuracy is not affected by an unfavorable placental location or a high maternal body mass index [7].

The gold standard treatment of invasive placentation is cesarean hysterectomy. The recommended gestational age for planned cesarean birth and hysterectomy is 34–35 + 6/7 weeks of gestation [17]. Traditionally, a cesarean hysterectomy is performed with the placenta left in situ after the fetus is delivered. Delayed hysterectomy is another possible surgical treatment option for PAS, where a scheduled hysterectomy can then be arranged 3–12 weeks postpartum, giving the advantage of less blood loss since the uterine perfusion decreases after delivery [7]. Temporary blockage of internal iliac arteries before the cesarean section is also an efficient way of managing placenta previa–accreta, since it decreases the intraoperative blood loss and the need for blood transfusion, while it minimizes the need of emergency obstetric hysterectomy [18–21]. When fertility preservation is desired, conservative management can also be considered. The placenta is either entirely removed or the part of it that is adherent to the myometrium is left in situ, with the aim to preserve the uterus. A Bakri balloon can also be used in order to reduce bleeding when conservative management is performed [22].

## 2. Case Presentation

**Figure 1:**
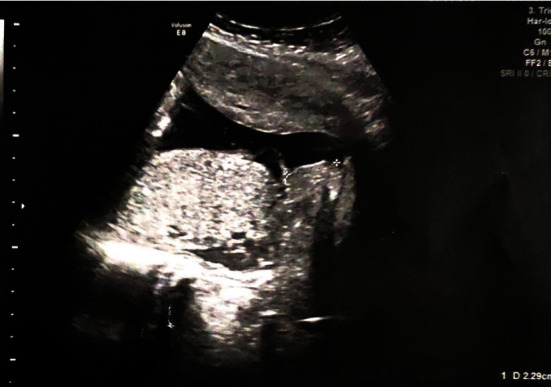
Transvaginal ultrasound indicates the leading edge of placenta at 2.29 cm from the internal os on the day the woman presented at the hospital.

A 32-year-old woman (G2, P0) had a cesarean section in her first pregnancy, due to failure of labor progress. Her child suffered from a prenatally undiagnosed tetralogy of Fallot and underwent an elective repair with open-heart surgery during the fourth month of his life. Unfortunately, he died 3 years later due to right ventricular hypertrophy. The patient had undergone dilatation and curettage 6 years ago, due to the presence of endometrial polyp and she was an occasional smoker. In her current pregnancy, the patient was inconsistently monitored and was admitted at 38 weeks of gestation to a regional hospital with contractions and scar tenderness. On her admission, she had no clinical symptoms besides minimal spotting, whereas the ultrasound demonstrated a viable fetus with normal amniotic fluid and biometrical data. At ultrasound it was evident that the edge of the placenta was covering the internal cervical os (Figure 1). Her blood test results were as follows: white blood cells 8.850/*μ*l, hematocrit (Hct) 32%, and hemoglobin (Hb) 10.5 g/dl. On examination, cervical dilation was 3–4 cm and the patient had three regular contractions, every ten minutes. Given the findings of clinical examination and her history, the need for an immediate delivery was discussed with the patient at which point she expressed her wish to maintain her fertility. It was carefully explained to the patient that this could be an option if bleeding could be controlled following the delivery of the baby. In a different case, however, hysterectomy could be the only option to save her life.

The on call obstetric team undertook the operation. Following general anesthesia and opening of the abdominal wall, the presence of several wide vessels in the area of the bladder was noted. Bladder displacement was executed followed by a transverse uterine section above the lower uterine part in order to avoid the placental bed. A healthy baby boy weighing 2,940 g was delivered. After an incomplete spontaneous placental detachment, most of the placental tissue was manually removed, whereas the attached segment was left intact into the uterus. During placenta extraction, blood loss volume was estimated at approximately 650 ml, and the plan for conservative management was maintained, since the patient was hemodynamically stable.

To control the bleeding, hemostatic sutures and two collagen sponges Tachosil were used and a Bakri balloon was inserted into the uterus and filled with 400 ml of normal saline. An abdominal drain was placed in the pouch of Douglas and the abdomen was closed with diligent hemostasis. The full blood count intraoperatively revealed an Hct of 27.7% and an Hb of 9.2 g/dl, and at the end of the operation the woman, who was hemodynamically stable, was transferred to the postpartum recovery room under close monitoring. Because continuous vaginal bleeding was observed, misoprostol 1,000 *μ*g was administered rectally. However, PPH could not be adequately controlled. After informing the patient regarding the life-threatening situation, a decision for an emergency hysterectomy was taken.

Within 30 minutes the woman was transferred again to the surgery room and under general anesthesia an emergency operation took place. Intraoperatively, the blood pressure had dropped to 65/35 mmHg and the patient's pulse raised up to 150 bpm. The peritoneum was opened and as soon as the surgical team assessed the bleeding as life-threatening, blood transfusion was commenced immediately. Bilateral ligation of iliac arteries followed by hysterectomy with bilateral salpingo-oophorectomy was performed. In order to manage adequate hemostasis, each bleeding point was clamped minimally with a mosquito Kelly's forceps, grabbed with another mosquito Kelly's forceps, and then was ligated with 2-0 silk suture. The post-operative blood results showed Hct 16.1% and Hb 5.3 g/dl. The patient was hemodynamically unstable, and for this reason abdominal packing was applied to prevent hypothermia, coagulopathy, and acidosis, and common complications of uncontrollable bleeding. The abdominal wall was closed layer by layer and the woman was transferred intubated to internal care unit. The second operation lasted 140 minutes and the estimated blood loss was 8.100 ml. The patient received in total 18 units of red blood cells, 10 units of fresh frozen plasma, and 9 units of platelets. The uterus was sent for histopathological examination, which confirmed the diagnosis of PAS. The placenta had indeed invaded the myometrium without affecting the serosa of the uterus.

Three days later, being in good condition with normal vital signs, she underwent a second look laparotomy to remove the packs. The bleeding had stopped completely and the woman was hemodynamically stable, therefore, she moved to the obstetric department, from which she was discharged the following day in a satisfactory condition. Forty days postoperatively, she returned to the hospital for a typical follow up and no complications were observed.

## 3. Discussion

While infrequent in the past, PAS is becoming more and more common nowadays and is recognized as a major cause of maternal morbidity and mortality. The main risk factors are a previous cesarean section in combination with placenta previa, therefore, the increased incidence reflects the worldwide increase in the number of cesarean sections performed [3]. After a previous cesarean section, it is more common that a PAS disorder occurs in an anterior placenta. However, PAS with posterior placental location has been described in the literature and it is associated with delayed diagnosis, surgical complications, assisted reproductive technology, and lower numbers of prior cesarean deliveries relative to anterior location [23]. Additional risk factors that should draw the attention of the clinicians are previous operations that affect endometrium, such as curettage, in vitro fertilization, high maternal age, and smoking [7–9]. Early diagnosis is key for optimized outcomes since women with PAS should be treated by a multidisciplinary team at a level III or IV center. Because PAS can be fatal, hysterectomy is the most common treatment solution [24].

Conservative management should be considered only when intraoperative findings indicate that a hysterectomy is likely to be difficult, leading to a massive hemorrhage that may be minimized if the placenta is left in situ, or when the woman is reluctant to lose fertility [7].

A useful method for controlling hemorrhage in abnormal placentation deliveries is positioning of balloon vascular catheters since it can reduce intraoperative blood loss and avoid a hysterectomy [20–23]. This technique should be performed by an experienced team and unfortunately it is not uncommon for a regional hospital in the country to lack obstetricians with adequate training to carry out balloon catheterization.

If the conservative approach fails an emergency obstetric hysterectomy is the only solution to control the PPH and save the woman's life [25]. During the consent, it is crucial for the patient to understand the risks that come with either conservative management or hysterectomy. If the patient is hemodynamically stable, time should be given to her to decide after consulting with her immediate environment. This study highlights the need of a highly experienced health care team accustomed to the management of placenta accreta must be present in every hospital since early intervention can save a woman's life. Training of obstetricians and gynecologist should be modified and adapted to such kind of emergency situations. Clearly, the absence of an experienced team, which will perform an emergency obstetric hysterectomy can lead to the loss of woman's life [26].

## Data Availability

The data used to support the findings of this study are included within the article.
